# Varieties

**Published:** 1856-09

**Authors:** 


### VARIETIES.

"DIED, CORNELIUS BOGART, at his residence, 126 Bleecker-street, New York, on Monday, August 11th, 1856, of Dysentery, having passed four score years. It is said that he left his family well provided for, and that, until his fatal illness, he had never known a day's sickness. He was always a hale, hearty man, and continued in the practice of his profession (Law) until the last week of his life."

This is an instructive narrative, showing that a man may live to a good old age, in a large city; may enjoy vigorous and uninterrupted health until the day of his death, and may secure a steady, honorable prosperity. Why should not this be the rule, instead of the rare exception?

THE FLY PLAGUE.—Cover the openings of unclosed windows and doors with a net made of white thread, the meshes being about an inch in diameter, but the light must enter the room from one side only. This was made known by *Spence* twenty years ago. This keeps outsiders out; if you want to persecute them further, a Chinese Linden, or Lime tree before your door will poison a peck daily. If both these are impracticable, keep every room in your house, dining room, kitchen and all, clean, dry and darkened. Flies have a perfect antipathy against clean houses. Flies revel in filth, the world over.

EGGS are good for invalids sometimes, and always for healthy people. A tea spoonful of Cayenne Pepper given to a dozen hens, with their food, every other day, winter and summer, will nearly double the daily yield of eggs. This same Capsicum, at meals, is far better for the human stomach than brandy, better for the debilitated than any "*tonic*," drops or bitters, ever swallowed.

